# Empirical validation study and psychometric evaluation of the properties of the populist attitudes scale for the portuguese population

**DOI:** 10.1186/s40359-023-01118-1

**Published:** 2023-03-31

**Authors:** Filipe Falcão, Carlos Jalali, Patrício Costa

**Affiliations:** 1 Life and Health Sciences Research Institute (ICVS), School of Medicine, University of Minho, Largo do Paço, 4700-000 Braga, Portugal; 2 PT Government Associate Laboratory, ICVS/3B’s, Braga/Guimarães, Portugal; 3 Research Unit on Governance, Competitiveness and Public Policies, Department of Social, Political and Territorial Sciences, University of Aveiro, Aveiro, Portugal; 4 Faculty of Psychology and Education Sciences, University of Porto, Porto, Portugal

**Keywords:** Populism, Voter attitudes, Measuring populism, Personalisation of politics, Psychometrics

## Abstract

**Background and objective:**

Recent developments in Europe and Portugal provide a fertile ground for the rise of populism. Despite the growing interest in the topic, there is no reliable tool to gauge Portuguese citizens’ populist attitudes to date. The Populist Attitudes Scale (POP-AS), developed by Akkerman et al. [1], is one of the best-known instruments for measuring populist attitudes. However, no version for use in the Portuguese population is available. This paper describes the psychometric validation of the POP-AS for the Portuguese population.

**Methods:**

Trustworthy measures of validity suggested by Boateng et al. [2] to address the psychometric features of the POP-AS were approached. A robust psychometrical pipeline evaluated the reliability, construct validity, cross national/educational validity, and internal validity of the POP-AS.

**Results:**

The Portuguese version of the POP-AS exhibited sound internal consistency and demonstrated adequate properties of validity: a one-factor model was obtained, revealing evidence of construct validity; invariance was ensured for education and partially ensured for the country; All the items of the POP-AS revealed relatively good values of discrimination and contributed adequately to the total score of the scale, ensuring evidence of internal validity.

**Conclusion:**

Psychometric analysis supports the POP-AS as a valid and reliable instrument for measuring populist attitudes among Portuguese citizens. A validation framework for measurement instruments in political science was proposed. Implications of the findings are discussed.

**Supplementary Information:**

The online version contains supplementary material available at 10.1186/s40359-023-01118-1.

## Introduction

The emergence of populism in Europe and other regions over the last two decades has primarily been seen as a serious threat to European and global politics, especially in the wake of the 2008 financial crisis [3, 4]. This phenomenon began to arouse academic interest in Europe with the electoral success of populist parties, such as the *National Front* in France to the *Forza Italia* in Italy [1, 5].

Understanding populism, not least as it potentially contributes to democratic backsliding, is perhaps the most significant challenge faced by political scientists and democracy researchers nowadays [6]. One key element is assessing the demand side of populism: i.e., what constitute populist attitudes at individual level that can then potentially translate into support for populist politicians and parties.

To date, different strategies for gauging populist attitudes have been proposed [7, 8]. The Populist Attitudes Scale (POP-AS), developed by Akkerman et al. [1], is one of the best-known instruments for measuring support for populism. It is a six-item self-report questionnaire that focuses on core features of populism. The first four items express viewpoints on representative government, such as the assertions that there is a gap between voters and politicians and that elected officials do not adequately represent the needs of the people. The latter two depict moral aspects of the struggle between the elite and the general public. Although this instrument has been used in numerous cross-sectional studies aimed at assessing populist attitudes in a number of European nations [8, 9], it has never been applied or validated in Portugal.

This paper evaluates the psychometric properties of the POP-AS [1] in order to validate it within the Portuguese population. The article is designed as follows. First, we attempt to define populism, comprehend the reasons for its emergence, and examine how it affects voting. Second, we discuss the instruments that have been developed to gauge populist attitudes and fundamental measurement issues in political science. Finally, considering the Portuguese political landscape, we discuss the process for validating the POP-AS scale for the Portuguese population and provide a framework for the validation of measurement instruments in political science.

In the present paper, a cross-sectional study was designed using a sample of Portuguese respondents. Reliability and validity properties of the POP-AS were evaluated based on the following statistical pipeline: (i) item-total correlation, split-half reliability, and Cronbach’s alpha were conducted to test for internal consistency; (ii) confirmatory factor analysis was conducted to test for construct validity; (iii) Multi-group invariance testing within a sample of respondents from fourteen countries in Europe was used to ascertain evidence of cross-national and cross-educational validity; (iv) Item response theory (IRT) analysis was employed to determine evidence of internal validity.

## Background

### The “hour of populism”

Given the frequency and intensity of its occurrence in virtually every nation with a constitutional democracy, populism has gained prominence in today’s politics [10]. However, despite the increased interest, this phenomen still presents a conceptual challenge to academics [11]. Being neither an ideology nor a political system, populism is a nebulous concept that refers to a type of collective action intended to seize power [12]. Unlike other political ideologies, it is based on a crude moral logic with few obvious political implications, offering instead a simplistic critique of the power structures rather than a comprehensive understanding of society [6]. This purported ambiguity stems not only from the concept itself but also from the role that context plays in defining the ideological stances of populist actors. Since context significantly influences the focus of the populist actor (e.g., globalization, immigration, imperialism, Islam, etc.), one cannot consider populism to exist on its own, as it typically aligns with opposing ideologies like (neo-)liberalism, the extreme right, and socialism [1, 13].

Despite the debate, three central tenets of populism have gained widespread acceptance: (i) a belief that people and elites are two separate yet related groups (*anti-elitism*); (ii) the instigation of a morally driven, antagonistic confrontation between these two parties (*Manichean*); and (iii) a belief in the people as a moral sovereign and the need to reinstate the purportedly stolen people’s sovereignty [7, 14]. In light of these pillars, one may treat populism as a loosely set of ideas concerning the transformation of the democratic principles of the majority and the people through a leader representing it and an audience legitimizing it in a manner intended to elevate one group of the people as opposed to another [10].

Understanding populism, and the impact of these phenomena on factors such as participation and representation, is one of the most crucial tasks of political scientists today [11, 15]. With the global ascendence of populism showing no signs of abating, there has been an explosion of research combining academic work on American and comparative politics and encouraging dialogue among political scientists, economists, and sociologists [15].

More recently, academics have started quantifying populism at individual level using opinions or attitudes [1, 8]. There are at least two reasons for this approach. First, one must consider that populism distinguishes itself from a mere discontent and disenchantment with the political system by engaging in a type of politics of hope: i.e., the hope that ordinary people and the politicians may succeed where established parties and elites have failed [7]. Second, because the votes that populist parties receive do not necessarily reflect populist attitudes in the electorate. As Spruyt et al. [7] note, voters cast their ballots based on variety of considerations, requiring a distinction between populist vote and populist attitudes.

### Measuring the support for populism as an attitude

Different measures have been proposed to tap into the concept of populism and to measure populist attitudes and support at individual level [16]. Among them, the POP-AS, developed by Akkerman et al. [1], is one of the most comprehensive and supported approaches for measuring support for populism [7]. It is a self-report questionnaire for completion by individuals of voting age that aims to capture populism’s ideology and views of democracy, particularly its stress on the will of the people and the gap between the masses and the elite. The questions focus on the three core features of populism: the sovereignty of the people, anti-elitism, and the Manichean division [1].

Although this scale is widely used in studies on populism, its use is typically restricted to the mere translation of the original items [7, 17]. Consequently, no analysis of the instrument’s psychometric properties is conducted to test whether it can be applied to the target population. To our knowledge, few studies [1, 7, 8] focused on the validation of the psychometric properties of the POP-AS. The same applies to the other scales that assess the populism phenomena. This might be due to the dearth of empirical data guiding researchers in selecting survey questions [8]. Since most scale development studies in political science are at least partly driven by empirical data (and since the survey and questionnaire design have become central pieces to an increasing number of studies) [8], there is a need for guidelines that can assist researchers in conducting validation procedures, not only focused on populism but also political science in general, that can demonstrate that instruments measure what they were intented to measure [18].

This paper seeks to provide a framework for the validation of measurement instruments in political science by validating the POP-AS scale for the Portuguese population. With regard to populism, Portugal was for many years seen as an outlier [19], with some going so far as to describe the country as being almost immune to radical right populism [20]. However, the recent emergence of the populist radical right Enough! (*Chega!*) has changed this picture [21]. After winning a single seat in 2019 parliamentary elections, the year it was established, *Chega!* won 7.2% of the vote in the 2022 elections, becoming the third largest party in parliament. The rapid growth of this party, in a context hitherto seen as inauspicious to populism, makes the assessment of populist attitudes among the Portuguese all the more relevant.

To date, the reasons behind Portugal’s support for the far-right have not been thoroughly examined due to lack of data [22]. This highlights the need for instruments capable of measuring populist attitudes among the Portuguese. The present study constitutes the first attempt to validate an instrument capable of measuring populist attitudes in Portugal. In addition to validating the POP-AS, this paper provides a framework for the validation of measurement instruments in political science. It also adds to the literature by allowing future studies to use this instrument as tool to measure populist attitudes in Portugal [21].

## Statistical and psychometric methods

### Sampling and procedure

A representative sample took part in the current research. A quota sampling method considering age, sex, and region was used to gather the study sample. The data collection process was conducted by a panel company. Participants filled out an online survey after giving their consent in full. The questionnaire was intended to gather data regarding Portuguese voters’ voting patterns and populist attitudes. Original sample included 1343 Portuguese participants (N = 1343) from five Portuguese regions (36.4% North of Portugal; 23.2% Center of Portugal; 28.2% Lisbon; 4.5% Alentejo; 7.7% Algarve). Respondents’ ages ranged from 18 to 92 years (M = 41.3; SD = 13.1). Of these, 696 (51.8%) are females. 63% of the participants were highly educated. In addition to this sample, we used contemporary online survey data (N = 27,896) from 14 European nations (Portugal, France, Germany, Sweden, Poland, Italy, Spain, Greece, Switzerland, Austria, Denmark, Hungary, Netherlands, United Kingdom), with around 1993 participants per nation, to test for multi-group invariance. Data regarding non-portuguese participants was merged and gathered online through various datasets (e.g., Comparative Study of Electoral Systems; Longitudinal Internet Studies for the Social sciences; LIVEWHAT project – Living With Hard Times) made public for research. The sample of Portuguese citizens included in the multi-group invariance test was composed of the 1343 participants described above plus 1350 retrieved from a different survey conducted at the national level.

## Measures

### The POP-AS

Respondents of the POP-AS were asked to rate their agreement with six populism-related questions on a Likert scale ranging from 1 (“*I very much disagree*”) to 5 (“*I very much agree*”). The original six statements (Chronbach’s α = 0.82) form an additive index and load high on a single dimension (*populist attitudes*). As stated above, the first four items express views on representative government (e.g., “*The politicians in the Portuguese parliament need to follow the will of the people*”), including the notions that there is a gap between the electorate and the politicians and that elected officials do not truly reflect the people’s desire; the last two items reflect ideas about the Manichean division (e.g., “*Elected officials talk too much and take too little action*”).

### Translation of the POP-AS

The POP-AS was first translated from English into Portuguese following a back-translation process. The instrument was originally translated from its native tongue to Portuguese by a bilingual person. In a second phase, the translated draft was translated into its native language by a second bilingual individual who was not familiar with the instrument. The study authors assessed the items obtained – see Brislin and Freimanis paper[23] for a comprehensive overview of this process. No major issues regarding the items of the scale were found. The Portuguese version of the POP-AS is reported in the appendix of this manuscript (Cf. Appendix A).

### Statistical analysis and psychometric validation of the POP-AS

We approached trustworthy measures of validity suggested by Boateng et al. [2] to address the psychometric features of the POP-AS. A detailed explanation of the procedure follows.

### Internal consistency reliability

Internal consistency reliability was calculated using item-total correlation, split-half reliability, and Cronbach’s α. We first estimated internal consistency by calculating split-half reliability: 1000 split-half reliability tests were computed, of which the mean was used as the best estimate (> 0.70: acceptable). Second, we estimated the item-total correlations for all the items in the POP-AS (r > 0.30: acceptable) [24]. Finally, we calculated Cronbach’s α coefficient of the POP-AS (> 0.70: acceptable). Through these tests, one can assess the degree to which the set of items in the scale co-vary relative to their sum score [2, 25, 26].

### Evidence for construct validity: factor structure analysis

Construct validity of the POP-AS scale was assessed through Confirmatory Factor Analysis (CFA). The Kaiser-Meyer-Olkin (KMO) test for sampling adequacy and the Bartlett sphericity test were used to assess the suitability of the factor solution. The Chi-square test [27], the Root-Mean-Squared Error of Approximation (RMSEA) [28], the Comparative Fit Index (CFI) [29] and the Standardized Root Mean Square Residual (SRMR) [30] were used as measures of model fit.

### Evidence for cross-national/cross-educational validity: multi-group invariance testing

Measurement invariance evaluates the psychometric equivalence of a construct across groups or across time, which is relevant when instruments are used in cross-national surveys [8, 31]. In rating scales such as the POP-AS, it is important to rule out the possibility that individuals with an identical ability level (θ) on a latent construct have a different probability of giving a certain answer to an item depending on the group they’re included [32]. If the instrument is noninvariant, it is not possible to assess whether discrepancies between groups are due to actual differences in the latent trait or the result of different reaction patterns across the groups, let alone different understandings of the concept [8]. Consequently, an appropriate comparison of a construct between groups depends first on ensuring equivalence of meaning of the construct [31].

Three kinds of invariance are most often tested: configural, metric and scalar. Configural invariance means that all items are expected to load on the same latent factor; Metric invariance indicates that all factor loadings are forced to be equal across groups; Finally, scalar invariance indicates whether both factor loadings and indicators’ intercepts are invariant across groups [8]. In the present study, we performed a multi-group CFA in several steps to test whether the factor structure of the POP-AS was invariant across the country and education level. Following Fisher and Karl’s [32] work, we developed three different models: (i) first, we fitted a configural model (M1) by constraining one-factor loading for the POP-AS and one intercept to be identical for each group; (ii) Second, we created two nested models that sequentially constrained factor loadings (to test for metric invariance – M2) and item intercepts (to test for scalar invariance – M3) between groups [33]. Incremental and lack-of-fit indices were assessed to probe for invariance. Invariance was evaluated using the |CFI| > 0.9 and |SRMR| and |RMSEA| < 0.08 criteria [32]. Changes in |ΔRMSEA| and |ΔCFI| < 0.01 were also used to test for invariance. The measurement is considered noninvariant if the more limited model fits worse than the configural (i.e., there is variation in how the items or scale works across groups). However, if the constrained model does not have a significantly worse fit, the measurement is invariant (i.e., the indicators capture the latent variable in a similar way across groups) [8].

### Evidence for internal validity: item response theory analysis

In this study, the Graded Response Model (GRM) [34] was used for the analysis of the internal validity of the POP-AS. In addition to providing evidence about the internal validity of an instrument, the GRM is also able to provide evidence of construct validity [35]. The GRM is an Item Response Theory (IRT) model suitable for analysing the item responses characterized by polytomous categories [36–38]. IRT models determine the relationships between an individual’s underlying trait and the probability of endorsing an item using item and person characteristics [39]. Consequently, the probability of responding with a higher response option increases as the level of the underlying trait increases. Since these models assess the homogeneity of items using different external and internal conditions, they are proper tools for measuring the internal validity of an instrument through an item-level analysis of a measure [35, 40]. For an overview of IRT see [41].

The GRM specifies the probability of an individual to be qualified with a category *i*k or above, as opposed to being included in a lower category when the rating system has a minimum of three categories [42]. It includes information on how each item contributes to the ability of the measure to differentiate between individuals expressing different levels of competence [35]. Under the GRM, each item is comprised of *k* response categories, and their respective parameters are estimated for *k*-1 boundary response functions. Each function represents the probability of selecting any response option greater than the option of interest. Two parameters characterized each function: the discrimination parameter (*a*_*i*_) – which measures the magnitude of change of probability of responding to an item in a particular direction as a result of the trait level – and the difficulty parameter (i.e., threshold parameters) (*b*_*i*_) – which describes how high on a latent trait an individual needs to be in order to have a 0.5 probability of endorsing a given response category or a higher category [43, 44]. The GRM equation is as follows:

1 $$P_{ik\,(\theta ) = \frac{{exp\,[{\alpha _i}(\theta - {\beta _{ik}})]}}{{1 + \,exp[{\alpha _i}(\theta - {\beta _{ik}})]}}}^*$$

For Eq. 1, *P*_ik_ represents the cumulative probability of crossing threshold i or higher; *αi* refers to the parameter of the discrimination power of item i; *βik* refers to the parameter of difficulty for option k on item i; while θ refers to the estimation of the trait level of an individual. In this study, the Marginal Maximum Likelihood (MML) estimation method was used to estimate the standardized GRM. For an overview of the GRM, see Edelen and Reeve’s work [45].

### Software

R software (version 4.0.5), R Studio Desktop (version 1.4.1106) and R packages “*Psych*” [46], “*Lavaan*” [47], “sem” [48], “*ccpsyc*” [49], “*lsr*” [50], “*mirt*” [51], “sirt”[52] and “*eRm*”[53] were used to conduct statistical analyses. R package “*ggplot2*”[54] was used for graphical representations of the data.

## Results

### Internal consistency reliability

We obtained an average of all split-half reliabilities of 0.71 (SD = 0.05). Item-total correlations between the items were satisfactory and ranged from 0.3 (POP3) to 0.5 (POP4). The Cronbach α coefficient for the entire scale was 0.71. These values demonstrate good homogeneity across the items of the POP-AS scale [26].

### Evidence for construct validity: factor structure analysis

**Fig. 1:**
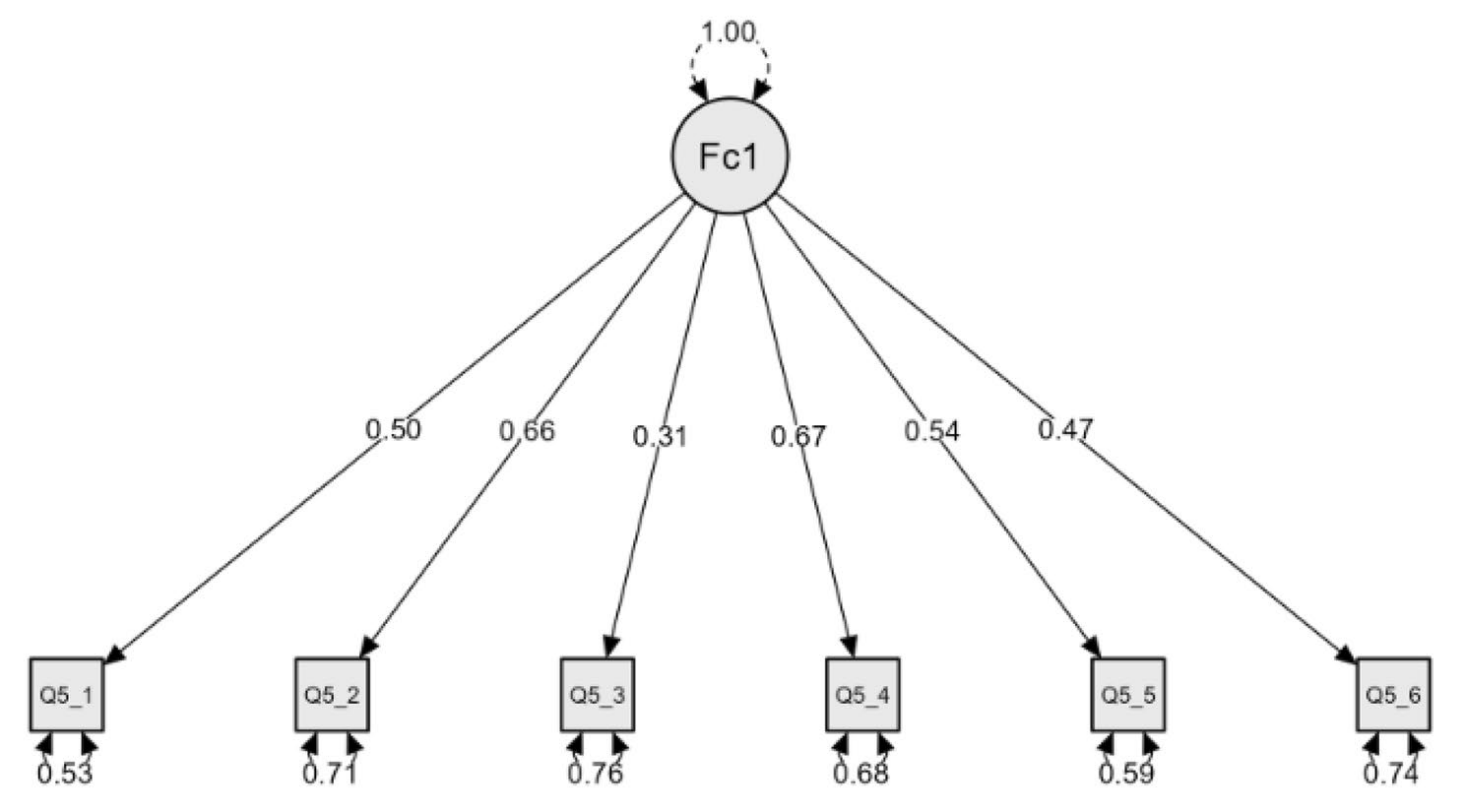
Path diagram of the structural equation model representing the POP-AS scale

Sampling adequacy of the factor solution was confirmed by the KMO test (KMO = 0.77) and Bartlett’s test of sphericity (χ^2^ = 1232; df = 15; *p* < 0.001). Results of the CFA revealed that the chi-square test statistic rejected the null hypothesis (χ^2^ = 84.8; df = 9; *p* < 0.001), which could indicate possible discrepancies or misfit; however, one should consider that this test is sensitive to the sample size [55]. Measures of fit revealed that the model fit the data relatively well, CFI = 0.94, SRMR = 0.04, and RMSEA = 0.08. Rules of thumb for cutoffs for an acceptable/good fit were CFI ≥ 0.90, SRMR ≤ 0.10, and RMSEA ≤ 0.08 [56, 57]. Figure 1 displays the standardized loadings for the one-factor solution (Cf. Table 1). No cross-loadings of less than 0.3 were found, reason why no items were removed [58]. Cronbach’s alpha for the single factor solution was acceptable (α = 0.71) [59].

**Table 1:** Factor structure of the POP-AS

Items	Loadings
POP1. The politicians in the Portuguese parliament need to follow the will of the people.	0.50
POP2. The people, and not politicians, should make our most important policy decisions.	0.66
POP3. The political differences between the elite and the people are larger than the differences among the people.	0.31
POP4. I would rather be represented by a citizen than by a specialized politician.	0.67
POP5. Elected officials talk too much and take too little action	0.54
POP6. What people call “compromise” in politics is really just selling out on one’s principles.	0.47

Cronbach α = 0.717

### Evidence for cross-national/cross-educational validity: multi-group invariance testing

The results for measurement invariance tests are presented in Table 2. For education, the configural model (M1) provided an acceptable fit (CFI = 0.98; RMSEA = 0.07), which indicates that the one-factor structure of the POP-AS is configurally invariant across all education levels. The Metric invariance model (M2) provided adequate fit and supported measurement invariance (CFI = 0.98; ΔCFI = 0.003; RMSEA = 0.07; ΔRMSEA = 0.006), which indicates that the items of the POP-AS are invariant and have the same meaning regardless of the education level. The Scalar invariance model (M3) provided support for measurement invariance (CFI = 0.98; ΔCFI = 0.003; RMSEA = 0.07; ΔRMSEA = 0.003), revealing that both factor loadings and item intercepts are invariant across different education levels [33].

For country, the configural model (M1) provided adequate fit (CFI = 0.96; RMSEA = 0.09), revealing that the one-factor structure of the POP-AS is configurally invariant across all the countries in study. Regarding Metric invariance (M2), we found out that the model reached only partial measurement invariance (CFI = 0.93; ΔCFI = 0.027; RMSEA = 0.09; ΔRMSEA = 0.004). Since the CFI value and changes in RMSEA were within acceptable values, one may partially state that the majority of the items of the POP-AS are invariant and have the same meaning across all countries [60]. This means that the correlations of the latent variable with other concepts can be compared across countries [61]. As partial metric invariance was supported, the next step was to test for scalar invariance. The Scalar invariance model (M3) failed to provide full support for measurement invariance (CFI = 0.79; ΔCFI = 0.14; RMSEA = 0.14; ΔRMSEA = 0.046). This means that factor loadings and indicator’s intercepts are noninvariant across groups [8]. As a result, one is unable to compare group means between respondents from different nations using the POP-AS.

**Table 2:** Reporting tests for measurement invariance

Group	Model	CFI	ΔCFI	RMSEA	ΔRMSEA
Education	M1	0.98	-	0.07	-
	M2	0.98	0.03	0.07	0.06
	M3	0.98	0.03	0.07	0.03
Country	M1	0.96	-	0.09	-
	M2	0.93	0.03	0.09	0.04
	M3	0.79	0.14	0.14	0.05

### Evidence for internal validity: IRT analysis

After ensuring all the IRT-assumptions (Cf. Appendix B) and appropriate model-data fit (Cf. Appendix C), the GRM was fit to our dataset. Since our sample size is considerable (N > 500), we were able to reliably estimate the discrimination and difficulty parameters of the POP-AS items based on the GRM [62]. Estimates of the a.i. ranged from 0.78 (POP3) to 1.60 (POP4) logits, representing moderate discrimination values (Cf. Table 3). Although the theoretical range for a.i. is -∞ to +∞, negative values, estimates below 0.24 and estimates larger than 3.00 are problematic [63]. Considering this interpretation, the estimates of a.i. suggest that the items of the POP-AS can differentiate respondents with a high trait and a low trait [64].

The *b*_*i*_ parameter represents the cut-points between the five-item categories. The range of b*i*1 to b*i*5 encompassed a negative-to-positive interval, which indicates that the items are more informative from the mildest to higher levels of populist attitudes [65]. The items covered a wide portion of the latent traits. The first threshold refers to a z-score value representing the probability of scoring in the first item response category (coded as 1) vs. scoring in a larger item response category (categories 2–5). *b*_*i*_ parameters ranged from − 6.08 to 2.64. The largest increment occurred between b_*i1*_ and b_*i2*_ (M = 2.25). This interval (b_*i1*_*-*b_*i2)*_ presented more variability than the others. The difference between these values can be interpreted as a measure of how easily a respondent may change their rating from one category to another. If the differences between the thresholds are small, small differences in the trait will lead to changes in the respondent’s ratings [35]. b_*i4*_ values were above the middle of θ. b_*i1*_ and b_*i2*_ values were situated below. b_*i3*_ values were situated approximately in the middle of the θ continuum. As expected, these values reveal that high trait levels are needed to have a probability of 0.5 to endorse higher categories of the items. Conversely, low trait levels are required to obtain a probability of 0.5 to endorse lower categories of the item.

**Table 3:** GRM item parameters estimations

	Slope	Threshold			
Item	*a*_*i*_ *(SE)*	b_*i1*_*(SE)*	b_*i2*_*(SE)*	b_*i3*_*(SE)*	b_i4_*(SE)*
POP1	1.43 (0.09)	-4.31 (0.35)	-2.18 (0.32)	-0.88 (0.03)	1.07 (0.03)
POP2	1.51 (0.10)	-2.27 (0.013)	-0.38 (0.09)	0.70 (0.10)	2.19 (1.03)
POP3	0.78 (0.07)	-6.08 (0.60)	-2.83 (0.42)	-0.98 (0.34)	2.11 (0.41)
POP4	1.60 (0.10)	-2.56 (0.14)	-0.87 (0.13)	0.39 (0.09)	1.73 (0.53)
POP5	1.53 (0.11)	-3.94 (0.30)	-2.00 (0.28)	-1.09 (0.25)	0.58 (0.23)
POP6	1.08 (0.08)	-3.87 (0.29)	-1.28 (0.19)	0.20 (0.16)	2.64 (0.73)

Considering item 1 (POP1) as an example, we see that the area under a standard normal curve to the left of -4.31 refers to the probability of endorsing category 1, and the area to the right is the probability of endorsing category two or higher. This result reveals that more people endorsed the first response category compared to the other categories. Item 3 had the lowest initial threshold value of -6.08, and item 2 had the highest initial threshold value of -2.27. The probability to the left of the threshold for item 2 was smaller than that of item 3. We found a lower response probability in the first category of this item, and this probability increased throughout the other categories. These estimates indicated that participants with low levels of populist attitudes were more likely to disagree with the items (i.e., lower scores).

In contrast, participants with higher levels of populist attitudes were more likely to agree with the items (i.e., higher scores). In the case of item 4, the most probable response for a trait score of -2.56 or below is *completely disagree*; if between − 2.56 and − 0.87, then the most probable response is *disagreed.* On the other hand, if the latent trait is between − 0.87 and 0.39, then the most probable response is agreed. If the trait is above 0.39, then the most probable response is *completely agreed*.

**Fig. 2:**
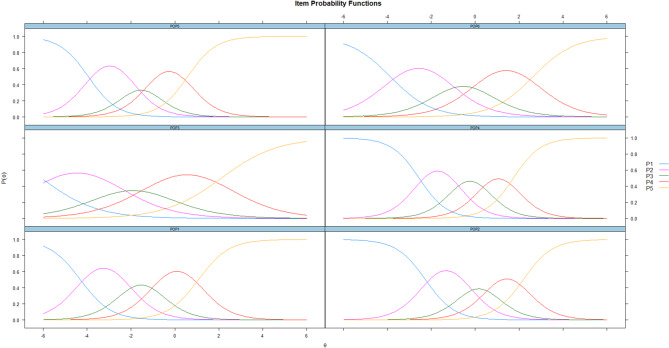
Probability functions for each item of the POP-AS scale

**Fig. 3:**
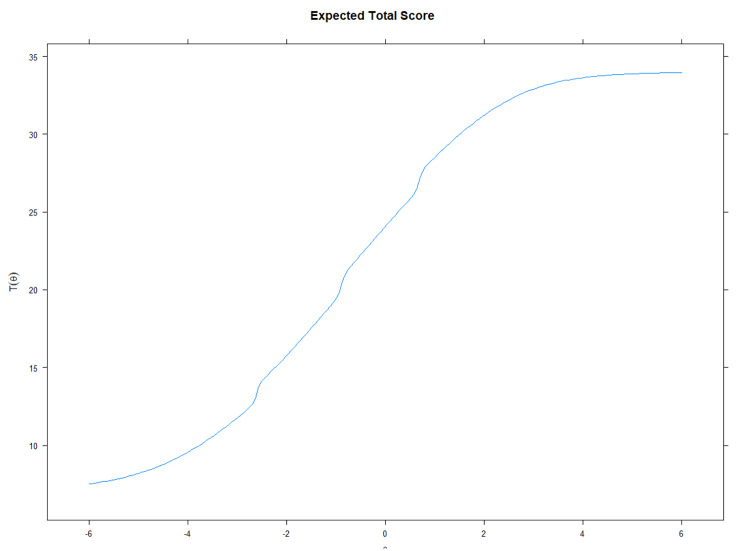
Expected total score function

Figure 2 displays the probability function curves for each item of the POP-AS. Each curve represents the probability of a respondent selecting/endorsing a category *k*, given the respondent’s θ represented by the plot’s x-axis (*M* = 0, *SD* = 1). Slopes of the curves for each item were determined by the a.i. parameter, and *b*_i_ values represented the intersection of each curve on the scale of the latent trait. High discriminating items (POP4 and POP5) had larger slopes and provided information regarding the latent trait in a narrow range of latent trait values. Moderate discriminating items (POP3) provided information across a wide range of the latent trait, representing broader ranges. The calibrated standardized scores for each item plotted as probability function graphs revealed that most items have their apex along the continuum in the positive half. This provides information regarding populist attitudes when there is an endorsement regarding the agreement characteristic. For example, item 1 is an endorsement of populist attitudes, as its apex for category 5 is situated in the positive half. This means that higher categories of the item are more likely to be endorsed by individuals with higher θ. Congruently, the expected total score function reveals that the score obtained on the scale increases according to the theta level, which aligns with what was said above (Cf. Figure 3).

**Fig. 4:**
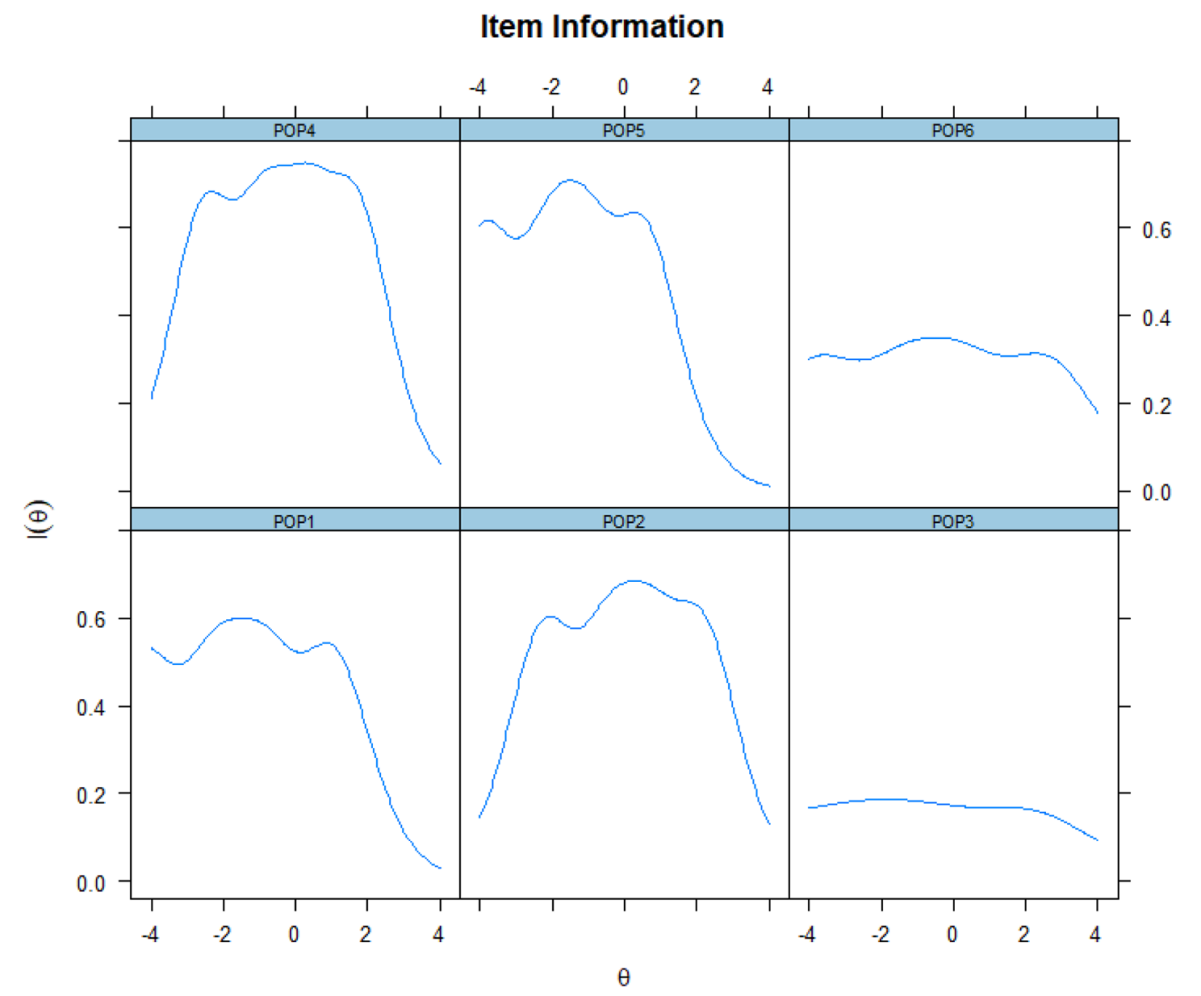
Item information functions

Reliability of the POP-AS was again evaluated using the GRM. Reliability (referred within IRT as “information”) differs across levels of the underlying trait being measured [66]. The item information function (IIF) evaluates how accurately each item measures different latent trait levels [44]. IIFs used to assess the informative and discriminative power of each item are presented in Fig. 4. Items 3 and 6 do not appear to be very informative.

**Fig. 5:**
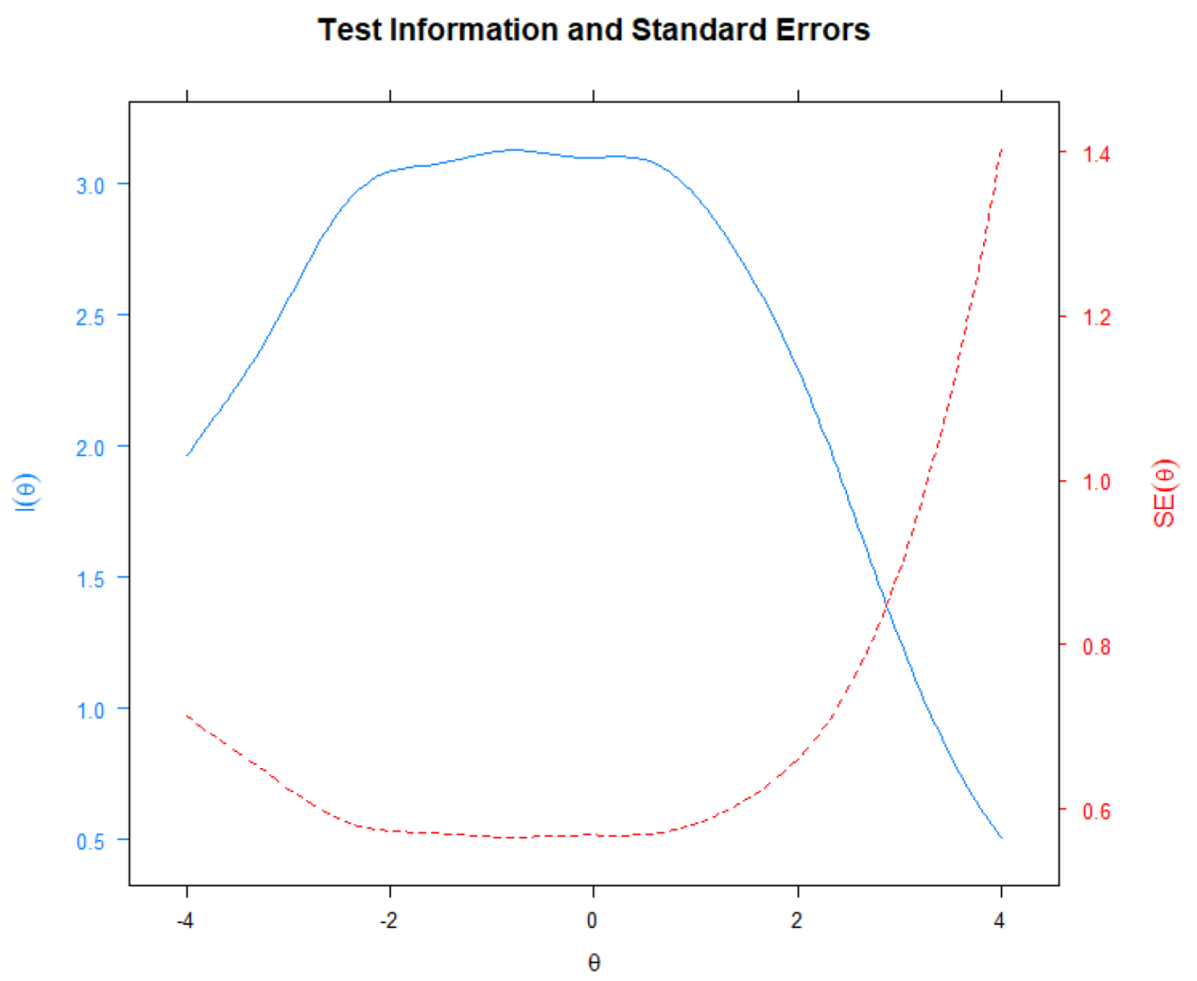
Test information function and measurement error curve

On the other hand, items 1,2,4 and 5 contribute the most to the scale. These IIF were then aggregated in a test information function (TIF) [67]. The TIF and the conditional standard error of the measurement function were used to assess the informative and discriminative power of the entire set of items (Cf. Figure 5). The TIF exhibited a wide estimated range. It peaked around − 1.5 and is shifted on the left side of the ability range, being more precise for low-intermediate ability levels. Figure 5 tell us that the POP-AS scale is more informative/ more precise for low and central scores of populist attitudes (θ levels between − 4.0 and 0). However, its precision decreases for subjects with high latent trait levels. Most of the information provided by the scale was above the mean θ of the respondents, with scores suggesting that the scale is better designed for respondents with lower θs (estimates between − 2.0 and 0).

**Fig. 6:**
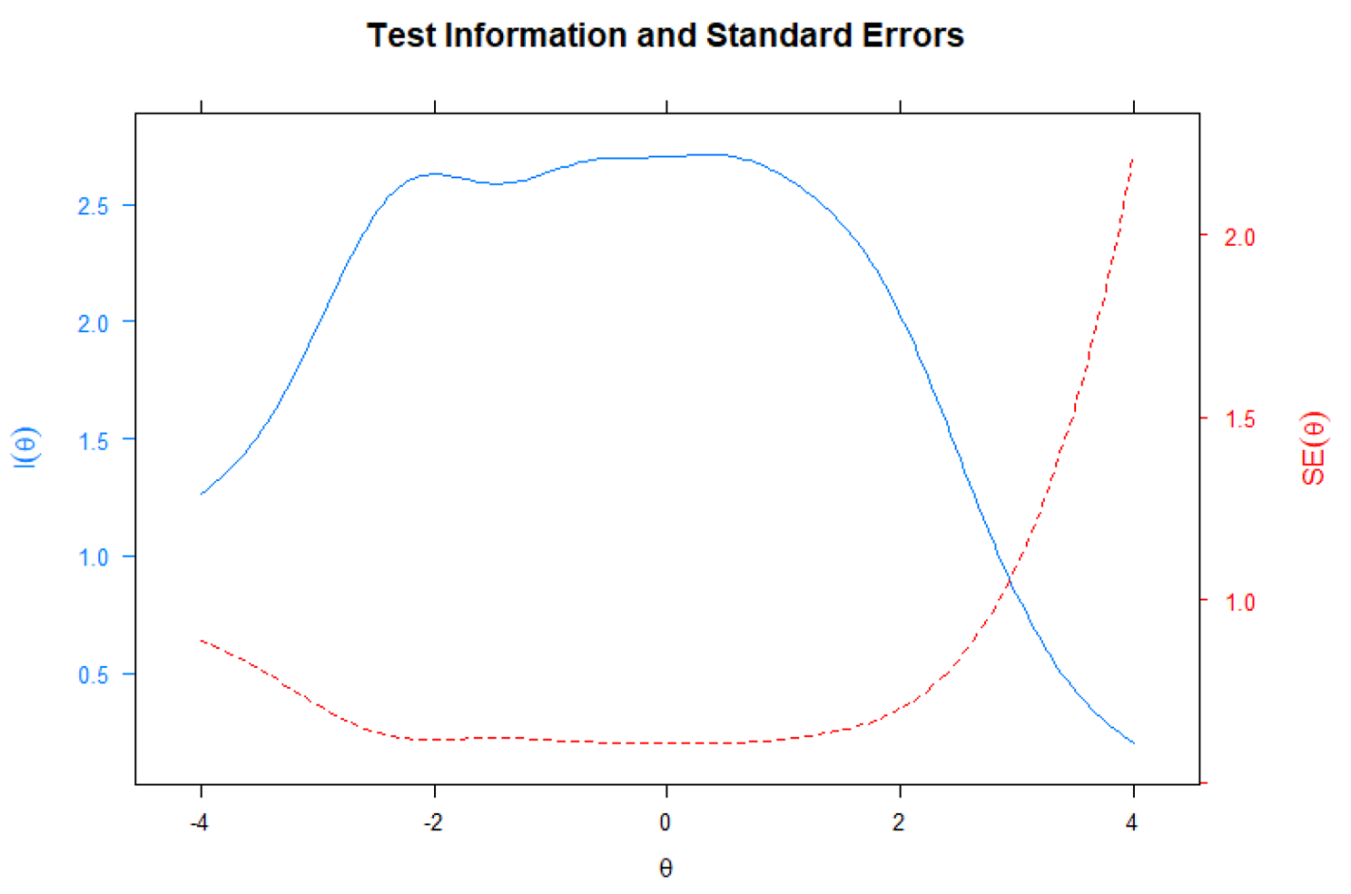
Test information function and measurement error curve (without items 3 and 6)

These results may suggest an eventual removal of items 3 and 6. However, the new TIF without these items did not translate into significant changes regarding the information provided by the new scale (Cf. Figure 6). Congruently, internal consistency analyses did not indicate significant reliability gains when these items were dropped. The alpha remained higher for the scale with all items (α = 0.72) than for the short form (α = 0.69). Additionally, we obtained estimates of the latent trait for each examinee using the full scale and the short form using the Maximum Likelihood Estimation method. Both forms provided similar ability estimates and high correlations among the latent trait estimates (r = 0.95), the reason why we chose to keep the items in question.

## Discussion

Over the past several years, there have been an unprecedented number of academic and public interest in understanding the factors leading to the rise of populism and how it affects democracy [68]. Numerous theories that attempt to explain the emergence of populism in modern democracies place particular emphasis on populist attitudes. While we must distinguish between populist attitudes and populist vote, the former are relevant in examining the growth and electoral potential for populists in Europe.

In this article, we provide a validation framework for evaluating the psychometric properties of the POP-AS[1] with the purpose of validating it for the Portuguese population. The outcomes were encouraging and demonstrate that the POP-AS is a valid and trustworthy measure for evaluating Portuguese populist attitudes. First, the scale items’ internal coherence was confirmed by the internal consistency metrics used. This means that the six questions of the instrument represented reliable arithmetic means to measure populist attitudes in the Portuguese population. It is also worth noting that the internal consistency test results of the original study were relatively better than ours. However, the values we obtained are still acceptable, which should not call into question the internal consistency of our version of the instrument.

Second, we determined the scale’s accuracy in measuring the intended outcome via factor analysis. The CFA supported the existence of one single dimension (Populist attitudes). All items composing the POP-AS were pertinent to and reflective of populist attitudes. This outcome is consistent with several research assessing the POP-factor AS’s structure, which represents evidence for construct validity [8]. It is important to note that the original study presented higher factor loadings for all items of the scale. Although our results are satisfactory, we believe that the cause of these lower factor loadings may be due to the fact that the Portuguese version of the POP-AS results from a translation process or from the characteristics of the samples themselves.

Third, we employed measurement invariance analysis to test for cross-national validity considering the country and educational level of respondents [8]. We found that the scale POP-AS exhibits configural, metric and scalar invariance across respondents’ educational levels. This proves that the POP-AS is a reliable measure to compare group mean scores of populist attitudes considering the respondents’ education levels. Regarding country, the POP-AS exhibited configural and metric (partially) invariance in all countries under study, but failed to reach scalar invariance. These results support the single factor structure found and informs us that most items in the scale are invariant (i.e., have the same meaning in all countries). It would be desirable to obtain scalar invariance as we obtained above for education. This outcome can be explained by a number of factors, including the history of the countries, ingrained cultural norms, governmental policies, and how the media reports on the nation’s politics, among others [69]. However, this result should not cast doubt on the POP-AS’s cross-national validity, since few cross-cultural studies test this level of invariance [70]. Most of the time, metric invariance is seen as a sufficient level to reach since, if attained, it does not skew regression estimates [8].

Fourth and finally, we evaluated the POP-AS for internal validity based on an IRT approach using the GRM. We found that the items of the scale captured adequate levels of information on a wide portion of the latent trait and successfully differentiated respondents with high and low levels of populist attitudes, ensuring the internal validity of the instrument. In general, the findings presented in this paper suggest that the POP-AS can be employed as a stable and valid measurement tool in future research to assess populist attitudes among the Portuguese population and even in comparative research.

The outcomes of this study have significant ramifications. Since the study of populism covers a variety of relevant topics within politics (such as foreign policy, party politics, political psychology and coalition formation), it is important for a country to be well-prepared and have at its disposal proper tools for the measurement of this phenomenon in all its citizens [71]. Atypical events like the COVID-19 outbreak, the Ukraine crisis, increased crime, and price inflation give populist forces more influence. Given these circumstances and the lack of a tool to gauge the populist attitudes of Portuguese citizens, it is vital to gather tools that can measure the populism phenomenon over time. Considering the results obtained, we believe that the POP-AS is a viable and trustworthy tool for assessing Portuguese populist attitudes.

The main strength of this research lies in validating a populist attitudes instrument through a strong framework that can serve as a guide for future validation studies in political science. The sample’s sizeable and representative nature lends credibility to the findings. The reviewed literature also enables a good overview of populism in Europe, not only in Portugal. However, it is also important to note that this paper has drawbacks. First, it does not compare the POP-AS to other populism scales published in the literature. Second, following other studies, we primarily derived our definition of populist attitudes from a supply-side theory, which may ignore factors unique to populist citizens [72]. Thirdly, it fails to implement a longitudinal method to test if the outcomes are sustained over time. These limitations can be addressed in future research on this topic.

## Conclusion

Due to recent events in Portuguese politics, the need to develop a measure capable of monitoring the populist attitudes of Portuguese citizens cannot be overstated. The POP-AS developed by [1] is a valid and reliable measure for evaluating populist attitudes in Portugal. A validation framework for measurement instruments in political science was also proposed.

## Electronic supplementary material

Below is the link to the electronic supplementary material.

Supplementary Material 1

## Data Availability

The datasets analysed during the current study are not publicly available but are available from the corresponding author on reasonable request.

## Abbreviations

POP-AS: Populist Attitudes Scale
IRT: Item response theory
PCP: Communist Party
BE: Left Bloc
CDS-PP: Centro Democrático e Social-Partido Popular
CFA: Confirmatory Factor Analysis
RMSEA: Root-Mean-Squared Error of Approximation
CFI: Comparative Fit Index
SRMR: Standardized Root Mean Square Residual
GRM: Graded Response Model
IIF: Item information function
TIF: Test information function
