# Supplementary material for: Empirical validation study and psychometric evaluation of the properties of the populist attitudes scale for the portuguese population
Source: BMC Psychol. 2023 Mar 31;11:93. doi: 10.1186/s40359-023-01118-1 (PMC10063943; doi:10.1186/s40359-023-01118-1)
Supplement: Supplementary file 1 — Supplementary Material 1 [file 40359_2023_1118_MOESM1_ESM.docx]

**Appendix**

**Appendix A: The Portuguese version of the POP-AS**

**Supplementary table 1.** The Portuguese POP-AS

| **Gostaria que me indicasse, por favor, o seu grau de concordância para cada uma das afirmações a seguir. Para responder utilize, por favor, uma escala de 1 até 5, em que 1 significa “discordo totalmente” e 5 “concordo totalmente”.** | | | | | |
| --- | --- | --- | --- | --- | --- |
|  | **1.Discordo totalmente** | **2.Discordo** | **3.Neutro** | **4.Concordo** | **5.Concordo totalmente** |
| **POP1.**    Os políticos no parlamento deviam seguir a vontade do povo. | 1 | 2 | 3 | 4 | 5 |
| **POP2.**    As pessoas, e não os políticos, é que deviam tomar decisões políticas mais importantes. | 1 | 2 | 3 | 4 | 5 |
| **POP3.**    As diferenças políticas entre a elite e o povo são maiores do que as diferenças entre o povo. | 1 | 2 | 3 | 4 | 5 |
| **POP4.**    Prefiro ser representado por um cidadão do que por um político profissional. | 1 | 2 | 3 | 4 | 5 |
| **POP5.**    Os políticos eleitos falam muito, mas fazem pouco. | 1 | 2 | 3 | 4 | 5 |
| **POP6.**    O que as pessoas chamam de fazer cedências na política é, na realidade, apenas vender os seus princípios. | 1 | 2 | 3 | 4 | 5 |

**Appendix B: IRT assumptions - strategies and results.**

IRT models require the following assumptions to be ensured: *unidimensionality* (i.e., one dimension explaining the covariance among items); *local* *independence* (i.e., absence of systematic conditional covariance among items) and *monotonicity between latent trait levels and true scores* (i.e., the need of the probability of endorsing an item to increase as the latent ability being measured levels increases) (Reeve et al., 2007). Strategies for assessing IRT assumptions and respective results are detailed below:

i) *Unidimensionality*: the unidimensionality assumption assures that the probability of an individual to respond in a particular direction is exclusively a function of one trait (i.e., populist attitudes) (Rubio et al., 2007). Factor analysis on the six items that make up the POP-AS scale revealed that the advised number of dimensions was one, ensuring this assumption.

ii) *Local independence*: the local independence assumption addresses whether the scale items are related to the dominant factor being measured and no other constructs (Lameijer et al., 2020). Since the assumption of unidimensionality was held, one can assume that the assumption of local independence was also met since the two concepts are strictly related through a single latent trait (Desjardins & Bulut, 2018; Lord, 2012). However, we have decided to ensure compliance with this assumption by evaluating the residual correlation matrix resulting from the single-factor CFA model. A value of 0.20 above the average residual correlation was considered for local dependence (Lameijer et al., 2020). As the average residual correlation for the CFA model was 0.0001, the critical value would be 0.20 as well. No residual correlations were larger than 0.2, suggesting local independence.

iii) *Monotonicity*: the monotonicity assumption refers to whether the probability of a response to the items increases with the increasing levels of the latent trait (Lameijer et al., 2020). Monotonicity was evaluated by fitting a non-parametric IRT model through Mokken scalling (Lameijer et al., 2020; Van der Ark, 2007). Scalability coefficients were above 0.30 for each scale item, ensuring the monotonicity assumption (Lameijer et al., 2020; Mokken, 1971). Monotonicity was secured through graphical visualisation of the probability functions for each item of the POP-AS scale (Cf. Figure 2). The probability of responding to an item was a monotonically increasing function of θ for almost all the items in the POP-AS scale, corroborating this assumption.

***Appendix C: Model-data fit***

*Model fit*

As there are no generally accepted goodness-of-fit measures of the GRM, we compared a common-slope GRM with an unconstrained GRM (Gray-Little et al., 1997). The p-value of the log-likelihood ratio test result was less than 0.001 (*p* < .001), rejecting the reduced model and favouring the unconstrained GRM. The corrected Akaike’s information criterion (AIC) and the Bayesian information criterion (BIC) values were also inferior for the unconstrained model (AIC= 20414, BIC= 20570; AIC_[c]_= 20477, BIC_[c]_= 20607) ensuring adequate fit of the GRM to our dataset. We applied the best fitting model to our POP-AS data to calculate item discrimination and threshold IRT parameters.

*Item fit*

We employed Pearson’s χ­^2^ statistics (S-χ^2^) to evaluate the fit of the POP-AS items to the GRM. For this method, significance tests can be used under the null hypothesis that the GRM fits the data adequately. We retained a nominal type I error rate of 0.001 for all analyses. Two items (POP1; POP3) were flagged as misfitting (*p* < .001). However, since it is not recommended to decide on the permanence/removal of items just based on fit statistics, and since there is no consensus on how to assess item fit, we decided to maintain these items in the next analyses (Cabrera-Nguyen, 2010; Desjardins & Bulut, 2018).

*Person fit*

To achieve person fit, participants' response patterns had to be aligned with the GRM. We used the standardized fit index (Zh) to ensure person fit (Drasgow et al., 1985). Results revealed that the Zh statistic was larger than -2 for most respondents, ensuring person fit (Cf. Supplementary figure 1).

**Supplementary figure 1.**  Person fit histogram.


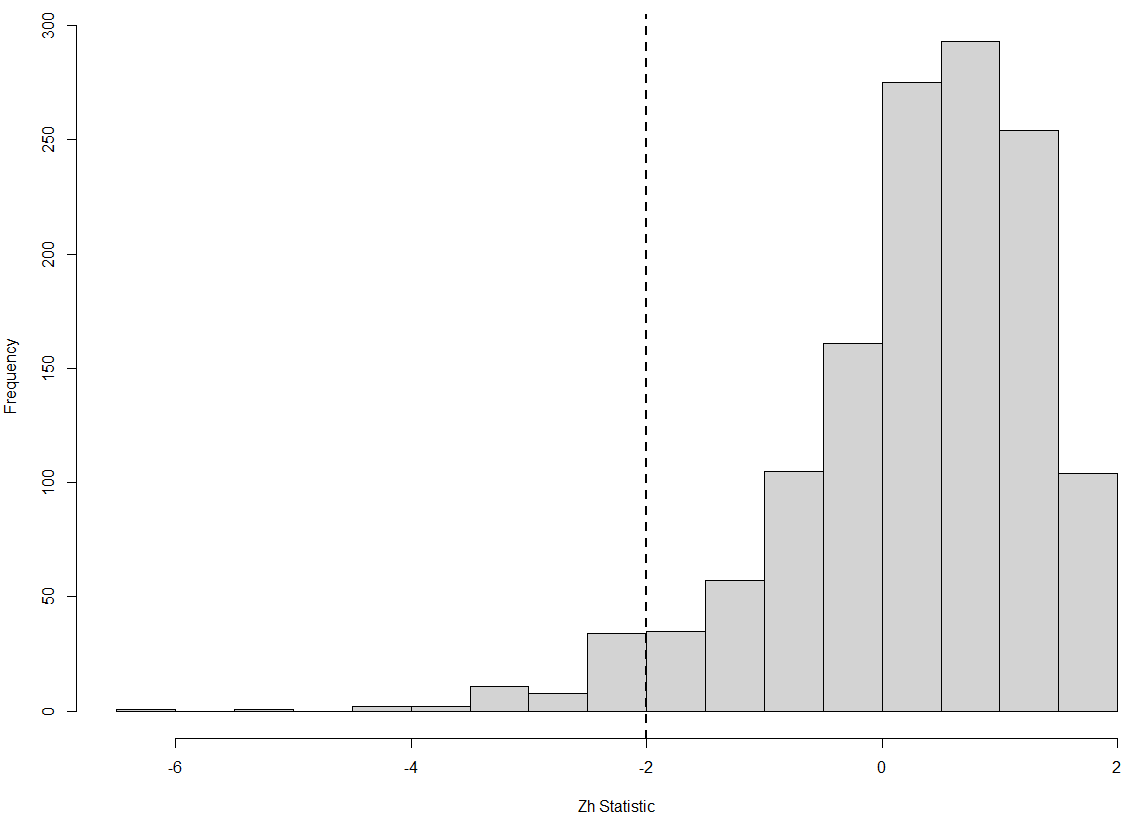


Note. Large negative values (e.g., Zh < −2) indicate person misfit. Large positive values (e.g., Zh > 2) reveal that the likelihood of the pattern of the responses is higher than the predictive likelihood of the GRM, disturbing latent estimation.
